# PP2A-B′ holoenzyme substrate recognition, regulation and role in cytokinesis

**DOI:** 10.1038/celldisc.2017.27

**Published:** 2017-08-08

**Authors:** Cheng-Guo Wu, Hui Chen, Feng Guo, Vikash K Yadav, Sean J Mcilwain, Michael Rowse, Alka Choudhary, Ziqing Lin, Yitong Li, Tingjia Gu, Aiping Zheng, Qingge Xu, Woojong Lee, Eduard Resch, Benjamin Johnson, Jenny Day, Ying Ge, Irene M Ong, Mark E Burkard, Ylva Ivarsson, Yongna Xing

**Affiliations:** 1 McArdle Laboratory for Cancer Research, Department of Oncology, University of Wisconsin at Madison, School of Medicine and Public Health, Madison, WI, USA; 2 Biophysics Program, University of Wisconsin at Madison, Madison, WI, USA; 3 Department of Chemistry—BMC, Uppsala University, Uppsala, Sweden; 4 Biostatistics and Medical Informatics, Wisconsin Institutes of Medical Research, University of Wisconsin at Madison, School of Medicine and Public Health, Madison, WI, USA; 5 Department of Medicine, Hematology/Oncology, UW Carbone Cancer Center, University of Wisconsin at Madison, School of Medicine and Public Health, Madison, WI, USA; 6 Department of Cell and Regenerative Biology, Human Proteomic Program, School of Medicine and Public Health, Madison, WI, USA; 7 Fraunhofer Institute for Molecular Biology and Applied Ecology IME, Project Group Translational Medicine and Pharmacology TMP, Frankfurt am Main, Germany

**Keywords:** PP2A-B′ holoenzyme, CIP2A, cytokinesis, midbody, centrosome, SLiMs

## Abstract

Protein phosphatase 2A (PP2A) is a major Ser/Thr phosphatase; it forms diverse heterotrimeric holoenzymes that counteract kinase actions. Using a peptidome that tiles the disordered regions of the human proteome, we identified proteins containing [LMFI]xx[ILV]xEx motifs that serve as interaction sites for B′-family PP2A regulatory subunits and holoenzymes. The B′-binding motifs have important roles in substrate recognition and in competitive inhibition of substrate binding. With more than 100 novel ligands identified, we confirmed that the recently identified LxxIxEx B′α-binding motifs serve as common binding sites for B′ subunits with minor variations, and that S/T phosphorylation or D/E residues at positions 2, 7, 8 and 9 of the motifs reinforce interactions. Hundreds of proteins in the human proteome harbor intrinsic or phosphorylation-responsive B′-interaction motifs, and localize at distinct cellular organelles, such as midbody, predicting kinase-facilitated recruitment of PP2A-B′ holoenzymes for tight spatiotemporal control of phosphorylation at mitosis and cytokinesis. Moroever, Polo-like kinase 1-mediated phosphorylation of Cyk4/RACGAP1, a centralspindlin component at the midbody, facilitates binding of both RhoA guanine nucleotide exchange factor (epithelial cell transforming sequence 2 (Ect2)) and PP2A-B′ that in turn dephosphorylates Cyk4 and disrupts Ect2 binding. This feedback signaling loop precisely controls RhoA activation and specifies a restricted region for cleavage furrow ingression. Our results provide a framework for further investigation of diverse signaling circuits formed by PP2A-B′ holoenzymes in various cellular processes.

## Introduction

Cellular processes and normal physiological functions require coordination of numerous covalent modifications, such as protein phosphorylation. The phosphorylation states of the over 700 000 unique serine/threonine/tyrosine phosphorylation sites detected in eukaryote cells [1, 2] are tightly and spatiotemporally controlled by hundreds of protein kinases and phosphatase complexes. While many kinases have well-characterized preferences for short linear motifs (SLiMs; 3–10 amino-acid stretches) for substrate targeting [3, 4], there is a scarcity of knowledge on substrate recognition of phosphatase complexes, which has been a critical barrier for understanding spatiotemporal control of protein phosphorylation in cellular signaling.

Protein phosphatase 2A (PP2A) is an essential and highly abundant serine/threonine phosphatase in eukaryotic cells, counteracting diverse kinases in cell growth, cell proliferation, apoptosis, cytoskeleton dynamics and stress response [5–7]. PP2A participates in such diverse cellular processes via formation of ~100 heterotrimeric holoenzymes, each containing a common core enzyme, a stable heterodimer of the scaffold A-subunit and catalytic C-subunit (PP2Ac) and a variable mutually exclusive regulatory B subunit from four families (B, B′, B″ and B‴). PP2A belongs to the phosphoprotein phosphatase (PPP) family of serine/threonine phosphatases (PP1–PP7). The SLiMs that target these phosphatases to their substrates have only been well described for PP1 and PP2B (calcineurin) [8, 9]. In case of PP2A, substrate recognition is largely controlled by regulatory subunits [10] but can potentially be controlled by both regulatory and catalytic subunits [11]. The regulatory subunits differ in their primary sequence and structure, which confer broad substrate specificity, cellular locations and tissue distributions [5]. The diverse regulatory subunits and holoenzymes present a huge challenge for elucidating PP2A substrates. The dearth of knowledge on substrate recognition by diverse PP2A holoenzymes has greatly hindered the dissection of PP2A function in diverse cellular signalings.

The B′ family of PP2A regulatory subunit consists of at least seven different members (α, β, γ1, γ2, γ3, δ and ε). A LxxIxE motif (where x is any amino acid) was recently suggested as preferred docking site for the B′α subunit, and a large set of ligands were identified through affinity purification coupled to mass spectrometry (AP-MS) and extensively validated by diverse strategies [12]. However, SLiM-based interactions are notoriously difficult to be discovered by AP-MS because of their often low-to-medium affinities, and they are often overpredicted by bioinformatics due to their limited footprints. We therefore took an unbiased approach to elucidate the SLiM-based interactions that target PP2A-B′ to its substrates, using proteomic peptide phage display (ProP-PD). ProP-PD is an emerging powerful technique dedicated to the discovery of SLiM-based protein–protein interactions powered by a combination of bioinformatics, phage display and next-generation sequencing [13–15]. Our recently created ProP-PD library tiles the intrinsically disordered regions of the proteome, and can be used to discover SLiM-binding preferences and targets of potential biological relevance for a variety of peptide-binding proteins [13–15].

Here we used ProP-PD to explore the SLiM-binding preferences of PP2A B′-family regulatory subunits and holoenzymes. We found a substantial overlap of identified ligands between the different regulatory subunits and holoenzymes, and confirmed that the most common SLiMs recruiting B′ subunits to substrates is ‘LxxIxEx’, with variations being allowed at position 1 and 4 ([LMFI]xx[ILV]xEx), and that the interaction is enhanced by negatively charged residues D/E or S/T phosphorylation at position 2, 7, 8 and 9. Through a combination of bioinformatics, biochemical and cellular biology approaches we identified intrinsic and phosphorylation-responsive B′-binding proteins that localize to the midbody and other cellular organelles, underlying tight spatiotemporal control of kinase-induced recruitment of PP2A-B′ holoenzymes to cellular supercomplexes. We elucidated the molecular basis of one such signaling circuit for Polo-like kinase 1 (Plk1)-facilitated binding of PP2A holoenzymes to Cyk4 in precise control of Cyk4 phosphorylation and epithelial cell transforming sequence 2 (Ect2) binding for proper RhoA activation and cleavage furrow progression during cytokinesis. Our results can serve to guide further investigations of PP2A-B′ holoenzyme functions in cell cycle and in broader cellular processes.

## Results

### ProP-PD selection to identify binding motifs for B′ regulatory subunits and holoenzymes

**Figure 1:**
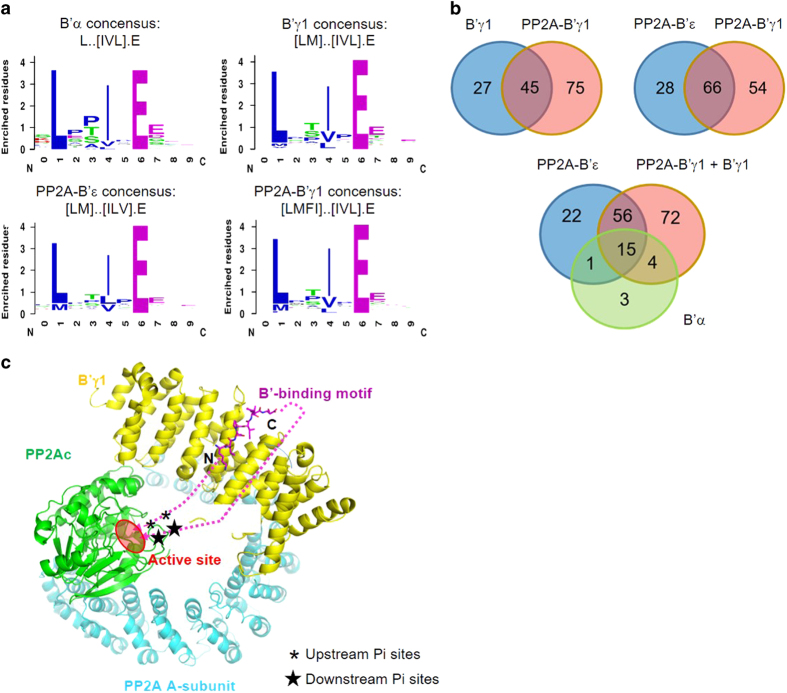
Identification of SLiMs containing peptides that bind to the B′ regulatory subunits and holoenzymes by ProP-PD selection. (**a**) Logos and consensus sequences of binding motifs for B′ regulatory subunits and holoenzymes identified by ProP-PD. Dots indicate that no strong preference of amino acids was found at the corresponding positions. (**b**) Venn diagrams indicating overlaps among peptides enriched from ProP-PD selection of B′γ1, B′α, B′γ1 holoenzyme (PP2A-B’γ1) and B′ε holoenzyme (PP2A-B’ε). (**c**) Structural illustration of substrate motif binding and placement of phosphorylation (Pi) sites surrounding the motif to the active site of PP2A-B′ holoenzyme. The structure of a consensus motif (magenta) from BubR1 bound to B′γ1 (yellow; PDB code: 5jja) was overlapped to structure of PP2A holoenzyme (PDB code: 2NPP). The dashed lines and stars stand for peptide fragments and phosphorylation sites upstream and downstream of the B′-interaction motif. N and C stand for N-terminal and C- terminal sides of the bound motif.

To elucidate the specificities of the B′ regulatory subunits, we first used glutathione *S*-transferase (GST)-tagged two remotely related B′ subunits (α and γ1; Supplementary Figure S1) as baits in selections against our ProP-PD library [13] that tiles the intrinsically disordered regions of the human proteome with 16-amino-acid peptides. The coverage of interacting SLiMs is ensured by an overlap of seven amino acids between flanking peptides. Enriched phage pools of the third day of selection were barcoded and analyzed by generation sequencing. We processed each data set of identified peptides by assigning a cutoff value of sequencing counts (cutoff=1), filtering out ligands that frequently occur in ProP-PD data sets and established consensus motifs for binding (Figure 1a) of remaining peptides using the SLiMFinder algorithm [16] to Lxx[IVL]xE for B′α and [LM]xx[ILV]xE for B′γ1. The consensus motifs were found in most peptides above the assigned cutoff value. Peptides with sequencing counts above the cutoff but lacking the motif may represent false positives, or hold alternative docking motifs. Peptides below the cutoff value containing sequences matching the motifs were included for the further analysis (Supplementary Table S1). After this analysis, 23 peptides were identified as binders for B′α and 72 peptides for B′γ1, with an overlap of 14 peptides between the two sets. The partial overlap between the ligand sets despite the similar binding SLiMs may indicate that we sample a subset of all potential ligands in each experiment and/or that B′α and B′γ1 exhibit minor differences in their binding specificities. The identified SLiMs are similar to the recently identified B′α-binding motif LxxIxEx [12]. Similar motifs were also previously identified in specific mitotic proteins BubR1 and Repo-Man for interaction with PP2A-B′α holoenzymes [17–19].

To establish the extent to which the specificities of the regulatory subunits are affected by the context of the holoenzymes, we performed phage selections against reconstituted holoenzymes PP2A-B′α, PP2A-B′γ1 and PP2A-B′ε, of which B′ε is most closely related to B′α among all known B′ family members (Supplementary Figure S1). The selection against reconstituted PP2A-B′α failed to produce binders. However, we successfully obtained 120 ligands for PP2A-B′γ1 holoenzyme and 94 peptides for PP2A-B′ε holoenzyme (Figure 1b; Supplementary Table S2). Although the much larger size of the holoenzymes might, in principle, cause steric hindrance, we found that the B′γ1 holoenzyme selections gave a larger number of ligands. We noticed that the free B′ subunits tend to form precipitates or aggregates *in vitro*, but the corresponding holoenzymes are more stable and soluble. Thus, the holoenzymes have more stable conformation and the binding sites for B′-binding motifs are most likely better presented in the holoenzymes, enabling the identification of more B′-binding motifs. We established the consensus motifs [LMFI]xx[IVL]xE for PP2A-B′γ1 holoenzymes and [LM]xx[ILV]xE for PP2A-B′ε holoenzymes (Figure 1a), which are similar to the motifs interacting with the regulatory subunits in isolation. Through a comparison of the identified peptide ligands, we found that the B′γ1 regulatory subunit and holoenzyme bind to almost all the B′α-binding peptides and the majority of peptides that bind PP2A-B′ε holoenzyme (71 out of 94; Figure 1b; Supplementary Table S2). The large overlap of the identified peptides between different B′-family members is consistent with the high sequence identity of their structural cores, where identical residues of B′ regulatory subunits are involved in motif binding [12, 20, 21].

We compared our ProP-PD-derived data set with the published data set on B′α-binding motifs identified by MS [12] to elucidate the overlap between the two approaches. Among the 23 peptide ligands obtained for B′α, four correspond to proteins previously found as PP2A B′α ligands by MS (Supplementary Table S2; BUB1B, RSF1, USP53 and FAM153), and additional four had been suggested as B′α ligands by bioinformatics analysis using the consensus motif, and retained after conservation filtering and removal of structured regions (CCNG2, WNK4, LPARA4 and PLCH2). Among the ligands identified for B′γ1 regulatory subunit and for PP2A-B′γ1 and PP2A-B′ε holoenzymes, 17 proteins had previously been suggested as B′α binders through AP-MS (Supplementary Table S2) [12], highlighting the overlapping binding specificities of the domains. In our merged set of B′ ligands, we identified 126 new B′ targets, among which 86 are in intrinsically disordered regions and not overlapping with any known/predicted domains.

Many of the identified sequences have known phosphorylation sites near the consensus sequence (Supplementary Table S2). On the basis of the mode of interaction of the motif from BubR1 and B′γ1 regulatory subunit [20], the upstream serine/threonine phosphorylation sites next to the motif can be readily placed to the active site of the PP2A-B′ holoenzyme, but not the downstream phosphorylation sites right next to the binding motif (Figure 1c). For example, pSer2151 (phosphosite.org) [3, 4] of centrosome-associated protein 350 (CEP350) is closely upstream of the B′-binding motif (identified peptide 2156-ERSRGS**L**ES**I**A**E**HVDA-2171; Supplementary Table S2) and are likely accessible to the active sites of PP2A-B′ holoenzymes, but the phosphorylation sites located closely downstream of the consensus binding motif, pS2172 and pS2174, likely not. The later assumption might have exceptions, however. As shown later, CEP350 is present in centrosome [22, 23] (Table 1), and its adjacent proteins at centrosome can recruit PP2A-B′ holoenzymes that might target those two sites in trans. Other phosphorylation sites further upstream and downstream of the consensus sequence of CEP350 might be targeted for dephosphorylation by PP2A-B′ holoenzymes as well, such as pT2104, pS2115, pS2127, pS2129, pT2138, pT2207 and pS2206 (Figure 1c) (phosphosite.org) [3, 4].

### Biochemical characterization of PP2A B′-binding motifs and global search for PP2A-B′ substrates

**Figure 2:**
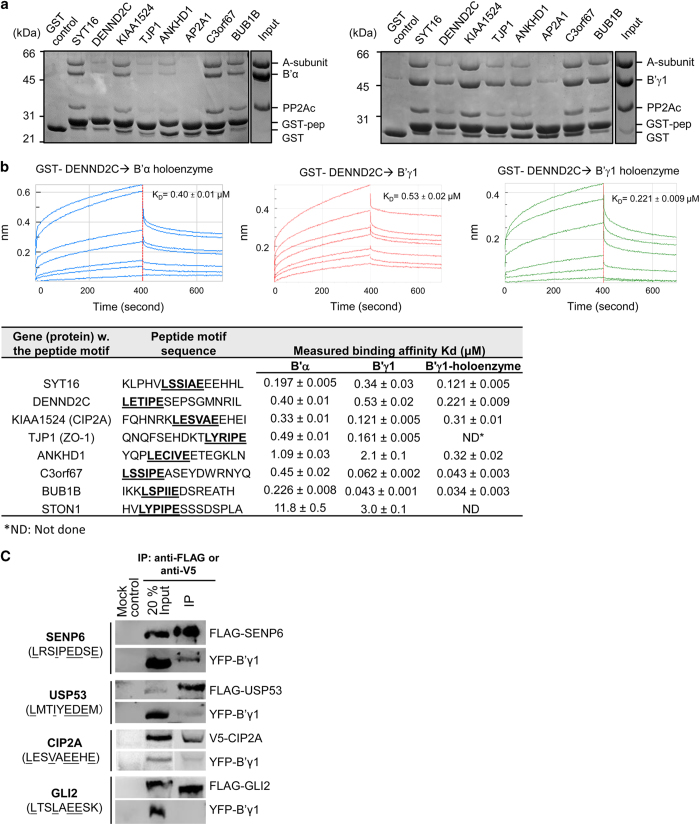
Binding assays of PP2A B′-binding motifs with B′ subunits and PP2A holoenzymes. (**a**) Pull-down PP2A-B′α (left) and PP2A-B′γ1 (right) holoenzyme via GST-tagged peptides suggested as PP2A B′ ligands by ProP-PD. Proteins associated with GS4B resins were examined by SDS-polyacrylamide gel electrophoresis and visualized by Coomassie blue staining. (**b**) Association and dissociation curves of binding between a representative GST-tagged peptide (DENND2C) to B′α (blue), B′γ1 (red) and PP2A-B′γ1 holoenzyme (green) detected by BLI. The calculated results for DENND2C and diverse other B′-interaction motifs identified by ProP-PD are summarized in the table below. (**c**) Co-immunoprecipitation (co-IP) of FLAG- and V5-tagged B′γ1 substrates identified by ProP-PD or bioinformatic prediction after co-transfection of FLAG-SENP6, FLAG-USP53, V5-CIP2A and FLAG-GLI2 with YFP-tagged B′γ1 in HEK293 cells. Lanes 1 and 2 show the blotting of indicated proteins of 20% amount of cell lysates used for co-IP.

To validate the identified ligands, we selected peptides that were found as binders in phage selections against both PP2A-B′γ1 holoenzyme and B′α regulatory subunit with high (SYT16 and DENND2C) and low (KIAA1524, C3orf67, and BUB1B, and STON1) sequencing frequency. We further selected ligands that were identified as ligands of B′γ1 or PP2A-B′γ1 holoenzyme (TJP1, ANKHD1 and AP2A1) but not B′α regulatory subunit. We expressed the selected peptides fused to GST and tested their interactions by GST pull down, showing that all tested ligands interact with PP2A-B′γ1 as well as PP2A-B′α holoenzymes, except AP2A1, which binds weakly to PP2A-B′γ1 and barely interacts with PP2A-B′α (Figure 2a). TJP1 and ANKHD1, which were selected for binding B′γ1, but not B′α, were found to interact with PP2A-B′α holoenzyme, albeit weaker than with the PP2A-B′γ1 holoenzyme (Figure 2a).

Next we systematically measured the on- and off rates, and calculated binding affinities of B′α and B′γ1 regulatory subunits and the PP2A-B′γ1 holoenzyme to the GST-tagged peptides. The study was performed using biolayer interferometry (BLI) with GST peptides immobilized on anti-GST sensors and titrated concentrations of B′ subunits and holoenzymes in the mobile phase (Figure 2b; example spectra for GST-DENND2C are shown, and results for all peptides are summarized in the table). The results suggest that the affinities are in the range of 0.03–11 μm, which is around one order of magnitude higher than the published isothermal titration calorimetry data [12], likely because BLI tends to overestimate the binding affinity. Nevertheless, the results showed that the peptides identified as B′γ1 ligands such as TJP1 and ANKHD1 also interact with B′α albeit with weaker binding affinities (Figure 2b), consistent with the results of the pull-down assay (Figure 2a). The minor differences between the binding affinities of the B′α and B′γ1 subunits support the notion that the B′ subunits have similar specificities, and that the partial overlap between the phage-derived ligands can be explained by minor differences in their interactions and the subsampling of a large set of available ligands. Previous studies showed that phosphorylation or negatively charged residues at positions 2, 7, 8 and 9 enhance B′ binding [12, 17, 21]. The measured binding affinities roughly reflect this correlation, except ANKHD1 (Figure 2b), which possesses perfect consensus residues at positions 1, 4 and 6, and three negatively charged residues at positions 2, 7 and 9. It is likely that residues at positions 3 and 5 or surrounding positions might affect binding as well, likely by alteration of the peptide conformation.

Phosphorylation-responsive B′ binding underlies an appealing mechanism for kinase-enhanced recruitment of PP2A-B′ holoenzymes in cellular signaling. We thus further examined whether pS/T at position 6 could replace the consensus ‘E’ residue. Three such peptides, LEPVRpSEE, LEPIRpSEE and LEPIRpTEE, were examined for their interaction with B′γ1. Even with three negatively charged residues at positions 2, 7 and 8, no interaction could be detected between B′γ1 and these phospho-peptides (Supplementary Figure S2). This observation is consistent with the recent observation that residue ‘E’ at positon 6 could not be replaced by the similarly negatively charged ‘D’ [12].

It is good practice to complement the ProP-PD experiments with bioinformatics as the phage display is a competitive approach that is ideal for providing information on specificity, rather than providing comprehensive information on all potential ligands available. In addition, the approach will not capture interactions that rely on post-translational modifications. To gain global insights into PP2A-B′ holoenzyme substrates and their potential responsiveness to phosphorylation, we therefore performed a bioinformatics search to identify such motifs in the disordered regions of the human proteome.

Hundreds of proteins were identified to harbor B′-binding motifs, with 1–4 D/E residues at position 2/7/8/9 that might partially correlate with the binding affinity. Even with exclusion of phosphorylation at position 6, many were found to harbor S/T at position 2/7/8/9 that can be potentially phosphorylated by kinases, and many of those potential phosphorylation sites have been identified in previous studies as indicated in phosphosite.org (Supplementary Table S3), underlying the potential of these targets to interact with PP2A-B′ holoenzymes upon phosphorylation.

To further validate the identified targets in mammalian cells, we selected the B′-binding proteins that were identified from phage selection (CIP2A) and from bioinformatic search of the human proteome (SENP6, USP53 and GLI2) for validation of their interactions with B′γ1 in mammalian cells. On the basis of the fitness of the target sequence to consensus logos and the number of negatively charged residues at positions 2, 7, 8 and 9, we predicted that CIP2A, CENP6 and USP53 are good binder to B′ subunits, while GLI2 is a weak binder. Consistently, we could detect the interactions of YFP-B′γ1 with CIP2A, SENP6 and USP53, but not with GLI2 by co-immunoprecipitation (Figure 2c).

On the basis of the crystal structures and previous and our data on B′-motif binding (Figure 2) [12, 17, 21], we further refined the bioinformatic search of human proteome for identifying B′-binding motifs. The binding pocket in B′ favors negatively charged residues and disfavors positively charged residues at positions 2, 7, 8 and 9. While the best consensus residues at positions 1 and 4 are L and I, respectively, changes of these two residues to other similar residues would reduce the binding affinity by 5- to 7-fold [12]. One replacement of residue at 1 and 4 to similar residues can be potentially supplemented by two or three negatively charged residues at positions 2, 7, 8 and 9 (Figure 2c). On the basis of these analysis, we applied the following rules in our search: (1) count the number of D/E and S/T, minus the number of K/R at positions 2, 7, 8 and 9, to obtain the number *N*; and (2) for positions 1 and 4, if both are the best consensus residues, the minimal value of *N* is 1; if one residue is replaced with a similar residue, the minimal value of *N* is 2; if both are replaced with similar residues, the minimal value of *N* is 4. The results of the improved search are provided in Supplementary Table S3.

### The mechanism of CIP2A in inhibition of PP2A-B′ holoenzymes

**Figure 3:**
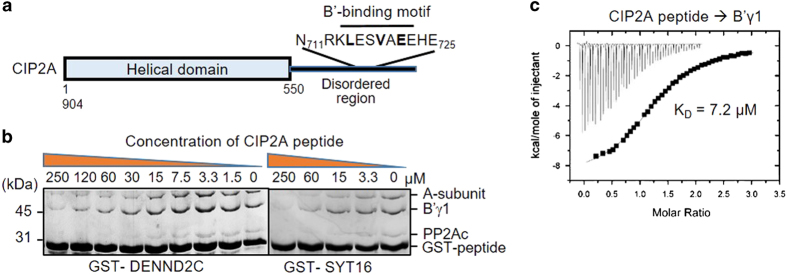
The interaction between CIP2A and PP2A-B′ holoenzymes reveals the mechanism of CIP2A in inhibition of PP2A-B′ holoenzyme function. (**a**) Schematic representation of protein domains of CIP2A and the location of its B′-binding motif. (**b**) Pull-down assays between GST-tagged B′-binding motif (GST-DENND2C and GST-SYT16) and PP2A-B′γ1 holoenzyme was blocked by increasing concentrations of synthetic peptides of the B′-binding motif of CIP2A. Proteins associated with GS4B resins were examined similar to Figure 2a. (**c**) Isothermal titration calorimetry measured the binding affinity between CIP2A synthetic peptide and B′γ1. The result showed a direct but weak interaction between CIP2A and B′γ.

A peptide of the KIAA1524-encoded protein CIP2A (the cancerous inhibitor of PP2A), 713- FQHNRKLESVAEEHEI-722, is among the common peptides identified by ProP-PD for B′α, B′γ1 and B′ε subunits and holoenzymes (Figure 1; Supplementary Tables S1 and S2). CIP2A was previously shown to block PP2A activity leading to enhanced cancer cell signaling such as those mediated by Akt, MYC and E2F1 [24–27]. The mechanism of CIP2A in PP2A inhibition, however, remained elusive. The identification of a B′-binding CIP2A peptide suggests that CIP2A might inhibit PP2A activity by competing with the substrate-binding site of the PP2A-B′ holoenzymes. The amino-acid sequence of CIP2A is predicted to contain an N-terminal helix domain (residue 1–550) followed by disordered region that harbors the B′-interaction motif (Figure 3a). While the N-terminal helical domain does not co-migrate with B′γ1 regulatory subunit over gel filtration chromatography (Supplementary Figure S3), the synthetic peptide NRKLESVAEEHE, representing the motif from CIP2A, blocks binding of PP2A-B′γ1 holoenzyme to GST-DENND2C or GST-SYT16 in a concentration-dependent manner (Figure 3b). We measured an affinity of 7.2 μm between the CIP2A peptide and B′γ1 by isothermal titration calorimetry (Figure 3c), which is comparable to the efficacy of CIP2A peptide in blocking holoenzyme–substrate interaction (Figure 3b), and confirms our earlier notion that the affinities measured by BLI (0.3 μm; Figure 2b) were overestimated. The relatively low binding affinity between CIP2A peptide and the B′ subunits is consistent with the fact that its cancer-promoting activity requires the presence of an increased level of CIP2A to outcompete interactions with substrates [24–27].

### Insights of bioinformatic-predicted targets of PP2A-B′ in cell cycle

Gene ontology term enrichment analysis of B′-interaction SLiMs suggested that PP2A-B′ holoenzyme substrates are involved in broad cellular and physiological processes and have links to diverse human diseases (Supplementary Table S4). Thousands of spatiotemporally tightly controlled protein phosphorylation events are dedicated to the proper progression of cell cycle through mitosis and cytokinesis [2, 28, 29], in which numerous kinases and phosphatases are precisely coordinated in dynamic cellular supercomplexes, such as the kinetochore, midbody and centrosome. A total of 229 proteins have been found to be associated with the midbody and are critical for cytokinesis, cell division and chromosome segregation [30–34], 11% of which are kinases [34]. To facilitate systematic understanding of how PP2A-B′ holoenzymes might specifically affect cell cycle and cytokinesis, we performed further bioinformatic analysis to identify substrates at distinct cellular supercomplexes, including kinetochore (k), telomere (t), spindle (s), centrosome (c) and midbody (m) (Supplementary Table S3). Many proteins at centrosome and midbody harbor intrinsic B′-interaction motifs with some important examples shown in Table 1; among the 10 proteins listed, 6 was identified by ProP-PD (Supplementary Tables S1 and S2). An even larger number of proteins at the centrosome and midbody potentially respond to phosphorylation for interaction with PP2A-B′ holoenzymes (examples shown in Table 2), many of which were not identified by ProP-PD. These include Cyk4 (also known as MgcRacGAP or RACGAP1), a centralspindlin protein located at midbody, a dense structure containing microtubules derived from the spindle midzone that is crucial for controlling cleavage furrow progression and cytokinesis [35].

All the examples in Tables 1 and 2 are further confirmed to be located in the disordered region based on secondary structural analysis using XtalPred (Supplementary Figures S4 and S5). These results suggest that PP2A-B′ holoenzymes are recruited by diverse centrosomal and midbody proteins, which might be highly responsive and enhanced by robust mitotic phosphorylation, presumably by Plk1 and other mitotic kinases. Thus, mitotic kinases and PP2A-B′ phosphatases might form diverse signaling circuits to precisely regulate a pool of phosphoproteins for precise control of mitosis and cytokinesis. Consistent with this notion, the signaling loop formed by Aurora B kinase and PP2A-B′ holoenzymes was found to control the length of spindle midzone by regulating KIF4A phosphorylation [36], and phosphorylation of KIF4A was recently shown to enhance its interaction with B′α [12].

### Biochemical characterization of Plk1-mediated phosphorylation of Cyk4 in B′ binding

**Figure 4:**
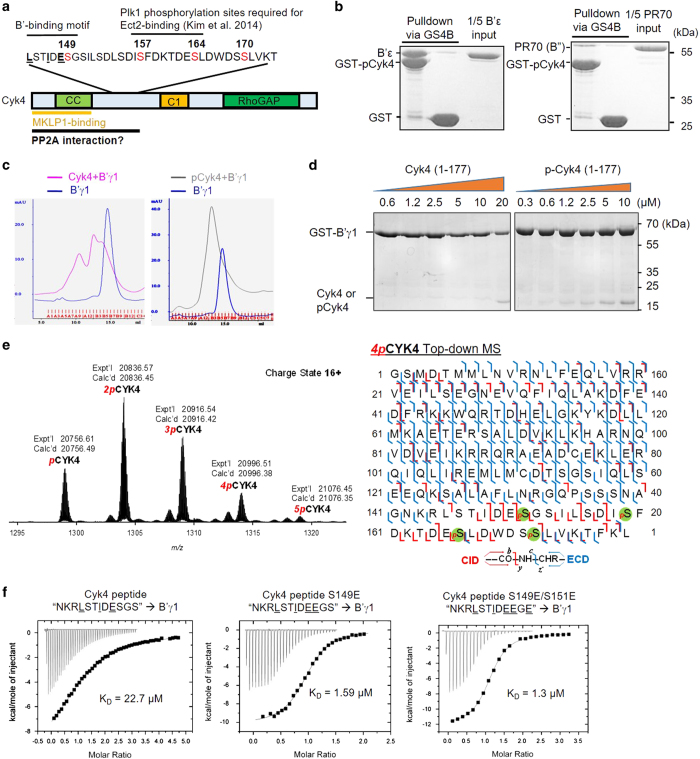
Plk1-mediated Cyk4 phosphorylation facilitates Cyk4 interaction with PP2A-B′. (**a**) Schematic representation of protein domains of Cyk4, and the locations of B′-binding motif, mitotic kinesin-like protein 1 (MKLP1)-binding region, the Plk1 phosphorylation sites (red) and Ect2-binding motif. Locations of coiled-coil (CC), C1 and RhoGAP domains on Cyk4 are indicated. (**b**) Pull down of GST-pCyk4 (1–177) with B′ε, and PR70 via GS4B resin. Proteins associated with GS4B resins were examined similar to Figure 2a. (**c**) Gel filtration chromatography of B′γ1 alone and its mixture with Cyk4 (1–177; left panel) and pCyk4 (1–177; right panel). B′γ1 co-migrates with pCyk4 (1–177), but not with Cyk4 (1–177). (**d**) Pull down of GST-B′γ1 with titrated concentrations of Cyk4 (1–177; left) and pCyk4 (1–177; right). The results showed that B′γ1 has a very weak binding with Cyk4 (1–177) while the binding is drastically increased upon Plk1-catalyzed phosphorylation (pCyk4; 1–177). (**e**) Top-down MS characterized the phosphorylation sites of Cyk4. MS shows differential phosphorylation of Cyk4 (left). The tetrakisphosphorylated Cyk4 was further identified by tandem MS (right). The sequence map of Cyk4 and its fragments identified by CID (red) and ECD (blue) were indicated. The four phosphorylation sites were localized to Ser151, Ser159, Ser166 and Ser172 based on 1 CID and 1 ECD spectra as highlighted. *p*, 2*p*, 3*p*, 4*p* and 5*p* represent mono-, bis-, tris-, tetrakis- and pentakis-phosphorylated CYK4, respectively. (**f**) Isothermal titration calorimetry measured the binding affinities between B′γ1 and original (left panel) or phosphomimetic Cyk4 peptides (S149E, middle panel; S149E/S151E, right panel).

To further demonstrate how mitotic kinases and PP2A-B′ signaling loops control cell cycle, we next examined how Cyk4 phosphorylation controls recruitment of PP2A-B′ to midbody and affects RhoA activation and cleavage furrow progression. Cyk4 and mitotic kinesin-like protein 1 form a hetero-oligomeric complex known as centralspindlin that has an important role in cytokinesis and the formation of the midbody [37]. Cyk4 contains a coiled-coil domain near the N terminus that is required for oligomerization and mitotic kinesin-like protein 1 binding, a long disordered region following coiled-coil that harbors a high density of phosphorylation sites, and a RhoGAP domain near the C terminus reversibly controls RhoA activation (Figure 4a). We previously showed that Plk1 catalyzes phosphorylation at multiple sites of Cyk4; two of the phosphorylation sites are crucial for recruitment of Ect2, a guanine nucleotide exchange factor required for activation of RhoA, cleavage furrow formation and ingression [38] (Figure 4a). The phosphorylation-responsive B′-interaction motif of Cyk4 (143-LSTIDESGS-151) predicted by bioinformatic search (Table 2) is located upstream of the Ect2-binding site, which harbors several potential Plk1 phosphorylation sites (Figure 4a). En route of our investigation, this motif was also suggested by another group as a B′-docking site, and phosphorylation at positions 2 and 7 was shown to enhance B′ binding [12].

To gain insight into how Cyk4 phosphorylation by Plk1 affects its binding to B′ subunits, we examined the interaction between Cyk4 and different PP2A regulatory subunits. Upon phosphorylation by Plk1, a Cyk4 construct (residue 1–177) containing the coiled-coil domain and a portion of the disordered region binds readily to B′ε, but not to PR70, a B′ family of PP2A regulatory subunit (Figure 4b). As shown in details below, this interaction was phosphorylation-dependent and was pertinent to different members of B′ family regulatory subunits. Both B′γ1 and B′ε co-migrate with phosphorylated Cyk4 (pCyk4; 1–177) over gel filtration chromatography, but not non-phosphorylated Cyk4 (1–177; Figure 4c; Supplementary Figure S6).

Next, we thoroughly examined Cyk4 phosphorylation sites by MS and the effect of each phosphorylation on B′ binding. Using titration pull-down assay, we showed that Cyk4 (1–177) barely interacts with GST-B′γ1 when the concentration of Cyk4 is below 20 μm (Figure 4d). Upon phosphorylation *in vitro* by Plk1, the interaction can be detected with Cyk4 concentration as low as 0.6 μm and the half maximum binding can be obtained with the concentration of pCyk4 (1–177) between 2.5 and 5 μm (Figure 4d). High-resolution top-down MS analysis [39, 40] revealed that Cyk4 is presented as mono-, bis-, tris-, tetrakis- and pentakis-phosphorylated proteoforms. No unphosphorylated CyK4 is detected (Figure 4e, left panel). The most abundant species are 2pCyk4 and 3pCyk4 that correspond to bis- and tris-phosphorylated proteoforms, respectively (Figure 4e, left panel). Further tandem MS with collision-induced dissociation (CID) and electron capture dissociation (ECD) of 4pCyk4 pinpointed the four sites that are phosphorylated, S149, S157, S164 and S170 (Figure 4e, right panel). Phosphorylation at these four sites was previously reported collectively, but not simultaneously, in different studies [41, 42]. This result is consistent with the fact that Plk1 phosphorylates Ser/Thr residues with the requirement of D/E/N at their −2 positions [43, 44]. As 5pCyk4 represents a minor species of the pCyk4 (Figure 4e, left panel), we concluded that the increase of binding affinity between B′ and pCyk4 is largely contributed by phosphorylation at S149. To corroborate with this notion, the binding affinity between B′γ1 and Cyk4 S149E peptide was measured to be 1.59 μm, 15-fold stronger than the native Cyk4 peptide and almost comparable to the peptide with double mutation S149E/S151E (Figure 4f). These collectively confirmed that pCyk4 interacts with PP2A-B′ holoenzymes via the identified B′-binding motif that is responsive to primarily the Plk1 phosphorylation site at S149.

### Plk1-mediated PP2A-B′ binding to centralspindlin regulates Ect2 recruitment, and the role of B′ in RhoA activation and cleavage furrow progression

**Figure 5:**
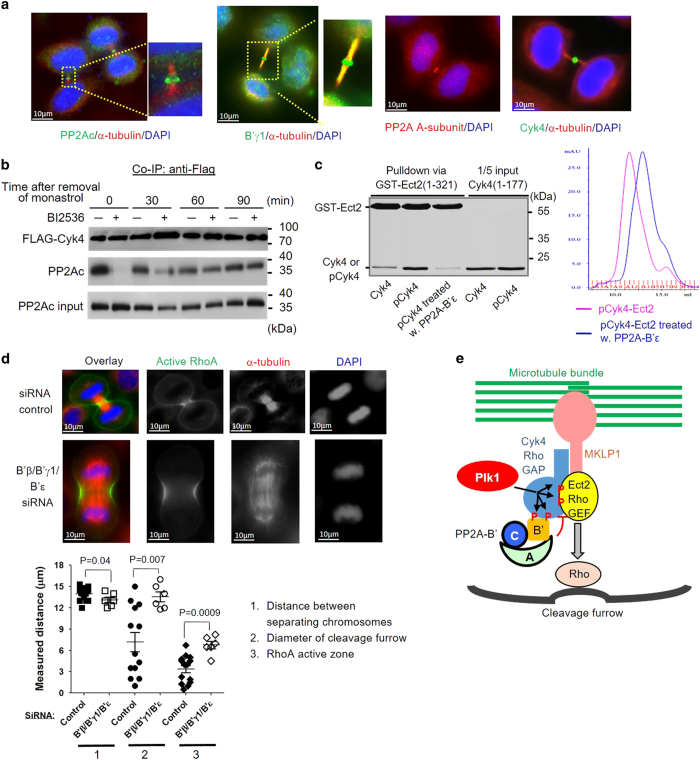
Plk1-dependent PP2A–Cyk4 interaction and the role of B′ in RhoA activation and cleavage furrow progression. (**a**) Immunostaining revealed the cellular localizations of PP2Ac, A-subunit and B′γ1 regulatory subunits at the dark zone and bulge region of the midbody, similar to Cyk4. (**b**) Co-immunoprecipitation (Co-IP) of FLAG-Cyk4 with PP2Ac in the presence and absence of PIk1 inhibitor (BI-2536) in the presence of recombinant V5-CIP2A. The interactions were examined at different time points (0–90 min) after removal of monastrol that synchronized the Hela cells at anaphase. PP2Ac was blotted to reflect the interactions between PP2A-B′ holoenzymes and Cyk4. (**c**) Pull down of pCyk4 by GST-Ect2 (left) and gel filtration chromatography of the pCyk4–Ect2 complex (right) before and after treatment by PP2A-B′ε holoenzymes. (**d**) Immunostaining to examine the cellular structures of RhoA activation zone, α-tubulin and dividing chromosomes after B′β/B′γ1/B′ε siRNA knockdown for cells at mitotic anaphase 24 h after siRNA transfection. Measurements of distance between separating chromosomes, diameter of cleavage furrow and the RhoA active zone for cells at mitotic anaphase 24 h after transfection of B′β/B′γ1/B′ε siRNA were shown below. Results were compared with cells transfected with control siRNA. (**e**) Cartoon illustrating the signaling circuit formed by Plk1 and PP2A-B′ holoenzymes in controlling Cyk4 phosphorylation and Ect2 recruitment at the midbody and subsequent control of RhoA activation and cleavage furrow ingression.

Consistent with the biochemical observations, PP2Ac, the scaffold A-subunit and B′γ1 regulatory subunits were found to be all localized at the dark zone and bulge region of the midbody, similar to Cyk4 itself, in HeLa cells (Figure 5a). To further support that Plk1-mediated phosphorylation is crucial for Cyk4–PP2A interaction, we showed that Plk1 inhibitor BI-2536, when applied to synchronized Hela cells at anaphase, blocked and delayed the interaction between PP2Ac and Cyk4 in the presence of recombinant expression of V5-CIP2A during anaphase (Figure 5b).

Next, we examined how PP2A-B′ holoenzymes affect the interaction between pCyk4 (1–177) and Ect2 (1–321). After treatment by PP2A-B′ holoenzymes, the pCyk4–Ect2 complex assembled after Plk1 phosphorylation failed to be pulled down together via GST-Cyk4 or co-migrate as a complex over gel filtration chromatography (Figure 5c), suggesting that PP2A-B′ holoenzymes would reverse Cyk4–Ect2 interactions.

PP2A-B′ holoenzymes was previously shown to control the length of spindle midzone by regulating KIF4A phosphorylation [36]. In light of the role of PP2A-B′ in regulating Cyk4–Ect2 interaction (Figure 5c), we extended this study by examining how B′ subunits affect RhoA active zone and cleavage furrow progression. Multiple B′ regulatory subunits, B′β/B′γ/B′ε, were simultaneously knocked down by siRNA as previously described [36], and the cellular structures of RhoA activation zone, α-tubulin and chromosomes were carefully examined by immunostaining 24 h after siRNA transfection for cells at mitotic anaphase (Figure 5d). While the distance between separating chromosomes for cells with B′β/B′γ/B′ε siRNAs was similar to the cells with control siRNA, the diameters of cleavage furrow and the size of RhoA active zone were distinctly different between control and B′β/B′γ/B′ε knockdown cells (Figure 5d). For control cells, the diameter of cleavage furrow (<1–15 μm) and the size of RhoA active zone (<1–7 μm) had broadly spread values (Figure 5d), reflecting an even distribution of the cells at different stages of cytokinesis. Cells with B′β/B′γ/B′ε siRNAs, however, were largely trapped at a stage with little ingression of cleavage furrow (12–15 μm in diameter), and diffused RhoA active zone (4–9 μm; Figure 5d).

Collectively, our results suggest an intriguing spatiotemporal coordination of Plk1 and PP2A during cytokinesis (Figure 5e), in which Plk1-mediated phosphorylation of Cyk4 recruits both Ect2 and PP2A-B′ holoenzymes; the latter in turn catalyzes dephosphorylation of Cyk4 and reverses Ect2 recruitments, which might have a crucial role in accurate spatiotemporal control of RhoA activation and cleavage furrow formation.

## Discussion

Complex PP2A oligomeric compositions and regulation allow PP2A to target numerous substrates and interacting proteins, making deciphering PP2A function in cellular signaling and diverse cellular processes a long-standing, daunting task. Here we used a recently developed phage library that displays a large proportion of the intrinsically disordered regions of the human proteome to chart the SLiM-based interactions of PP2A B′ regulatory subunits and holoenzymes. Through this unbiased approach, we confirm that the LxxIxEx is the preferred SLiM for interactions with different B′ regulatory subunits, and we find that the specificity is highly similar between the isolated regulatory subunits and the holoenzymes. In addition to the LxxIxEx-containing ligands recently identified as B′α by AP-MS [12], we identified more than 100 new putative substrates or binding proteins for B′ family, suggesting that ProP-PD is a powerful approach for rapid identification of SLiMs for interaction with diverse PP2A holoenzymes.

Our studies demonstrated that the interaction motifs identified for B′ family, although seemingly simple, provide several distinct mechanisms for regulating PP2A-B′ holoenzyme function: (1) substrate recognition (Figures 1 and 4); (2) competitive inhibition of substrate binding (Figure 3); (3) phosphorylation-facilitated recruitment of PP2A-B′ holoenzymes (Figures 4 and 5; Table 2); and (4) recruitment of PP2A-B′ holoenzymes to cellular supercomplexes, such as midbody, for tight control of protein phosphorylation in a spatiotemporally accurate manner (Figures 4 and 5; Tables 1 and 2). The B′ subunits of PP2A had been suggested to be important tumor suppressor proteins [45–49]. The identification of the common B′-binding motif in CIP2A reveals an important mechanism of CIP2A to compete with substrate binding in inhibition of the normal function PP2A-B′ holoenzymes and stimulation of cancer cell signaling [24–27]. It is important to mention that a weak interaction between B′ and the N-terminal helix domain of CIP2A was detected in a recent study [50], the resulting complex might not survive gel filtration chromatography and thus not detected in our study (Supplementary Figure S3). Thus, the B′-binding motif we identified here might serve as a secondary B′-binding site for CIP2A.

Phosphorylation-enhanced recruitment provides an elegant design of kinase-phosphatase signaling circuits, as reflected by the intricate control of Cyk4 phosphorylation and RhoA activation by Plk1 and PP2A-B′ holoenzymes (Figures 4 and 5). The signaling unit formed by Plk1 and PP2A-B′ holoenzymes on Cyk4 might be a representative mechanism for mitotic kinases and PP2A-B′ holoenzymes at mitosis and cytokinesis as diverse proteins at centrosome and midbody were found to harbor B′-interaction motifs (Table 1) and many are predicated to be responsive to phosphorylation (Table 2; Supplementary Table S3). It is important to mention that Cyk4 was also recently demonstrated to be a phosphorylation-responsive substrate of PP2A-B′α [12]. Comparing to this recent study, our advance here is threefold: (1) we demonstrated that Cyk4 interaction with PP2A-B′ family is drastically stimulated by phosphorylation by Plk1, and pinpointed the Plk1 phosphorylation site in Cyk4 that affects binding of PP2A-B′ holoenzymes; (2) we demonstrated that PP2A-B′ holoenzymes reverse the Plk1 phosphorylation sites in Cyk4 that are required for Ect2 binding, suggesting a concerted spatiotemporal control of kinase and phosphatase function in precise control of RhoA activation at the cleavage furrow; (3) to corroborate with the above observation, we showed that knockdown of B′ subunits altered the sharpening of RhoA active zone and cleavage furrow ingression.

Our global search of intrinsic and phosphorylation-responsive B′-interaction motifs with stratification of their cellular locations represent another powerful strategy for understanding robust signaling controlled by PP2A-B′ holoenzymes. Diverse B′-interacting proteins found at centrosome and midbody and their responsiveness to phosphorylation underlie mechanisms for spatiotemporally robust and accurate control of protein phosphorylation through mitosis and cytokinesis: the firing of mitotic phosphorylation by mitotic kinases also fires the recruitment of PP2A-B′ holoenzymes for precise, irreversible progression of cell cycle through mitotic exit and cytokinesis. Failure of proper progression through cytokinesis, such as those caused by B′ subunit knockdown (Figure 5), often lead to polyploidy and aneuploidy and loss of genome integrity, a phenomenon closely associated with cancer. Indeed, knockdown of B′ subunits had been shown to cause polyploidy [36]. This notion corroborates with the previous notion on the role of B′ subunits as tumor suppressors [45–49]. Our studies provide a platform for understanding PP2A-B′ holoenzymes in diverse other cellular processes.

Our study also corroborates the previous results that phosphorylation or negatively charged residues at positions 2, 7, 8 and 9 enhance B′ binding [12, 17, 21]. We showed that for targets that bear a B′-binding motif with perfect consensus residues at positions 1 and 4, their interactions with B′γ1 can be readily detected in mammalian cells if they contain two negatively charged residues at positions 2, 7, 8 and 9, such as SENP6 and USP53 (Figure 2c). For targets with residues similar to the consensus at positions 1 and 4, their interactions with B′γ1 can be detected if they contain three negatively charged residues at positions 2, 7, 8 and 9, as for CIP2A in both mammalian cells and *in vitro* (Figures 2 and 3), but failed to be detected if they only have one negatively charged residues at positions 2, 7, 8 and 9, such as GLI2 (Figure 2c). In case of Cyk4, which contains perfect consensus residues at positions 1 and 4, and no negatively charged residues at positions 2, 7, 8 and 9, one phosphorylation at position 7 appears to be sufficient to enable its interaction with B′ (Figure 4). These insights provide a knowledge framework for further characterization of B′-binding motifs and proteins in cellular signaling.

Finally, the multidisciplinary approaches and strategies evolved here can be taken for global identification and functional exploration of SLiMs for other PP2A holoenzymes in different families. Research along this line will culminate in an important knowledge framework on substrate recognition and interactomes of PP2A holoenzymes to enable convenient prediction and guide investigation of PP2A holoenzymes in diverse cellular signalings.

## Materials and methods

### Protein preparation

All constructs were generated using a standard PCR-based cloning strategy. Full-length B′α and B′ε were cloned in pQlink vector (Addgene, Cambridge, MA, USA) harboring a GST tag and a TEV cleavage site between the affinity tag and the protein. The proteins were overexpressed at 23 °C in *Escherichia coli* strain DH5α. The soluble fraction of the *E. coli* cell lysate was purified over GS4B resin (GE Healthcare, Boston, MA, USA), and further fractionated by anion exchange chromatography (Source 15Q, GE Healthcare) and gel filtration chromatography (Superdex 200, GE Healthcare). Expression and purification of full-length PP2A Aα, Cα, B′γ1 and PR70 (B″ family), and assembly of the PP2A core enzyme (Aα–Cα heterodimer) and PP2A-B′ holoenzymes followed procedures described previously [11, 51]. Expression and purification of GST-Cyk4 (1–177), Ect2 (1–321) and Plk1-phosphorylated Cyk4 (GST-pCyk4 or pCyk4), and the assembly of pCyk4–Ect2 complex were performed as previously described [38].

### Proteomic peptide phage display

The B′ regulatory subunits (B′α and B′γ1) and holoenzymes (PP2A-B′α, PP2A-B′γ1 and PP2A-B′ε) were used as bait proteins in selections against a phage library that displays 16-amino-acid peptides representing disordered regions of the human proteome [13]. The selections were carried out in semi-throughput format following a previously published protocol with minor modification [13, 52]. Proteins (20 μg in 100 μl Tris-buffered saline (TBS, 20 mm Tris-HCl and 150 mm NaCl, pH 7.4) were coated in 96-well Flat-bottom Immuno Maxisorp plates (Nunc, Roskilde, Denmark) overnight at 4 °C. In parallel, GST was plated in a pre-selection plate. The Maxisorp plates were blocked with 0.5% bovine serum albumin (BSA) in TBS for 1 h. The phage library [13] (~10^12^ phage particles in each well) in TBT buffer (TBS+0.05% (v/v) Tween20+0.5% (w/v) BSA) was added to the pre-selection plate for 1 h, transferred to the target proteins and were allowed to bind for 2 h at 4 °C. Unbound phages were removed by four times washing with cold wash buffer (TBS, 0.5% Tween-20) and bound phage was eluted by direct infection into bacteria by the addition of 100 μl of log phase (*A*_600_=0.8) *E. coli* Omnimax (Invitrogen, Carlsbad, CA, USA) in 2YT (16 g Bacto tryptone, 10 g Bacto yeast extract and 5 g NaCl, per liter water) to each well and incubation for 30 min at 37 °C with shaking. M13K07 helper phage (New England Biolabs (NEB), Ipswich, MA, USA) was added to a final concentration of 10^10^ phage per ml to enable phage production, and the cultures were incubated for 45 min at 37 °C with shaking. Eluted phages were amplified overnight in 10 ml 2YT supplemented with antibiotics (carbencillin and kanamycin) and 0.3 mm isopropyl-*β*-d-thiogalactoside. Bacteria were pelleted by centrifugation, and the phages were collected from the supernatant by addition of 2.5 ml 20% PEG800/0.4 m NaCl, incubation on ice for 10 min and centrifugation at 10 000 *g* for 10 min. The phage pellet was resuspended in 1 ml TBS and used for the next round of selection.

The progress of the selections was followed by pooled phage enzyme-linked immunosorbent assays. For these experiments, GST and GST-tagged regulatory subunits (10 μg in 100 μl TBS) were immobilized in a 96-well Maxisorp plate overnight at 4 °C. Wells were blocked with 200 μl TBS and 0.5% (w/v) BSA for 1 h at 4 °C and then washed four times with wash buffer. A volume of 100 μl of amplified out-phage pools of each day of selection were added and allowed to bind for 30 min at room temperature (RT) with shaking. The wells were washed four washes with 200 μl wash buffer, before incubation with horseradish peroxidase-conjugated anti-M13 antibody (GE Healthcare, 1:5000) in TBT buffer (100 μl) for 20 min at RT. Unbound antibody was removed by washing four times with wash buffer and one time with TBS. Bound antibody was detected by the addition of 100 μl of 3,3′,5,5′-tetramethylbenzidine peroxidase and substrate (1:1; KPL, New Delhi, India). The reaction was stopped after 5–10 min by the addition of 100 μl H_2_SO_4_ (0.6 m). The absorbance was measured at 450 nm.

As the above protocol failed to generate any ligands for the reconstituted holoenzymes (PP2A-′α, PP2A-B′γ1 and PP2A-B′ε) we established a modified protocol. GST-tagged reconstituted complexes (25 μg) or GST (20 μg) were allowed to associate with GSH-conjugated magnetic beads (20 μl, 1:1 bead/buffer slurry; Thermo Fisher scientific, Waltham, MA, USA) for 2 h, under gentle shaking at 4 °C. The beads were pelleted using a magnetic stand and the supernatant was removed. Before biopanning, the beads were washed four times with 1 ml TBS. Four successive rounds of phage selection and amplification were performed essentially following the same procedure described above, using the modified immobilization method and eluting bound phages by the addition of 100 μl of 100 mm HCl for 5 min at RT with gentle shaking. The acid-eluted phage pools were neutralized by the addition of 15 μl of 1.0 m Tris-HCl, pH 11.0 and used to infect *E. coli* Omnimax for amplification before the next round of selection.

Phage pools were barcoded for next-generation sequencing. Undiluted amplified phage pools (5 μl) were used as templates for 24 cycles 50 μl PCR reactions using a distinct set of barcoded primers (0.5 μm each primer) for each reaction, and Phusion High Fidelity DNA polymerase (NEB) with a maximum polymerase concentration. PCR reactions were supplemented with Gel Loading Dye Purple (6×; NEB) and separated on a 2.5% low-melt agarose (Bio-Rad, Hercules, CA, USA) gel stained with Roti-Safe GelStain (Carl-Roth, Karlsruhe, Germany). The DNA was visualized by ultraviolet light. The PCR products were extracted using the QIAquick Gel Extraction Kit (Qiagen, Hilden, Germany) according to the manufacturer with the following exceptions: (a) Gel extracts were resolved at RT; (b) DNA was eluted with 30 μl low Tris-EDTA buffer (Thermo Fisher Scientific, Waltham, MA, USA). Molarities of the eluted library DNA were determined on the 2100 Bioanalyzer using the High Sensitivity DNA Kit (Agilent, Santa Clara, CA, USA).

Template preparation was performed according to the manufacturer’s instruction using the Ion PGM Template OT2 200 Kit on the Ion OneTouch 2 System (Thermo Fisher Scientific, Waltham, MA, USA). A volume of 25 μl of 5 pm library DNA (1.25×10^−4^ pmol) were used in the template reaction. Sequencing was conducted on the Ion Torrent PGM sequencer using the Ion PGM Sequencing 200 Kit v2 and the Ion 314 Chip v2 (Thermo Fisher Scientific, Waltham, MA, USA) according to the manuals. Signal processing and base calling were done using the Torrent Suite Software (Thermo Fisher Scientific).

### GST-mediated pull-down and competitive binding assays

Approximately 10 μg of GST-tagged B′-binding motifs or GST-pCyk4 was bound to 10 μl of glutathione resin via GST tag. The resin was washed with 200 μl assay buffer three times to remove the excess unbound protein. Then, 10 μg of PP2A-B′ (PP2A-′α and PP2A-B′γ1) holoenzymes, B′ε or PR70 regulatory subunits was added to the resin in a 200 μl volume suspended in the assay buffer containing 25 mm Tris (pH 8.0), 150 mm NaCl and 3 mm dithiothreitol. The mixture was washed three times with the assay buffer. The proteins remained bound to resin were examined by SDS-polyacrylamide gel electrophoresis, and visualized by Coomassie blue staining. GST without the B′-binding motifs was used as control. The binding assay of B′γ1 with phosphorylated or non-phosphorylated Cyk4 was performed by similar procedure using 10 μg of immobilized GST-tagged B′γ1 to pull down titrated concentrations of phosphorylated or non-phosphorylated Cyk4. The competitive binding assay was performed similar to the procedure above, except that titrated concentrations of synthetic CIP2A peptide motif, RK**L**ES**V**A**E**EHE (0–250 μm), were mixed with 10 μg of PP2A-B′γ1 holoenzyme before addition to the GS4B resin with immobilized GST-B′-binding motifs (from DENND2C or SYT16). All experiments were repeated three times.

### Biolayer interferometry

BLI sensors immobilized by anti-GST antibody were activated by incubation with 300 nm GST-tagged B′-binding motifs, followed by incubation with 1 mg ml^−1^ BSA and 1 mg ml^−1^ GST, and wash by binding buffer containing 25 mm Tris (pH 8.0), 100 mm NaCl and 3 mm dithiothreitol, with 3 min each step. Seven sensors activated by GST-tagged peptide motifs were simultaneously dipped into seven wells containing the binding buffer control and increasing concentrations of B′α, B′γ1 and PP2A-B′γ1 holoenzyme (0–30 μm) to measure the on rate of the regulatory subunits or holoenzyme. After 400 s of binding, the sensors were dipped into binding buffer to measure the off rate for 300 s. Data collection and analysis were performed using ForteBio Octet RED96 and Data Analysis 9.0 (Pall Life Science, Port Washington, NY, USA), and the binding affinities were calculated based on the on- and off rates by fitting to the 1:1 binding model.

### Isothermal titration calorimetry

The binding affinities between B′γ1 and synthetic CIP2A and Cyk4 peptides (GenScript, Piscataway, NJ, USA) were determined by titrating 1 mm of peptides to 20–50 μm B′γ1 in 20 mm HEPES (pH 7.5), 200 mm NaCl using VP-ITC microcalorimeter (MicroCal, Malvern Instrument, Malvern, UK). The data were fitted with Origin 7.0 (MicroCal, Malvern Instrument, Malvern, UK) to calculate the equilibrium association constant.

### Migration and co-migration over gel filtration chromatography

A unit of 0.5 mg of B′γ1 or B′ε alone or their mixture with a stoichiometric amount of Cyk4 (1–177) or pCyk4 (1–177) were applied to gel filtration chromatography (Superdex 200, GE Healthcare) and the ultraviolet spectra of the B′ subunit alone and its Cyk4 mixture were overlaid to examine whether the B′ subunits co-migrate with Cyk4 or pCyk4. Likewise, the ultraviolet spectra of gel filtration chromatography of the pCyk4 (1–177)–Ect2 complex before or after treatment by PP2A-B′ε were compared to examined whether PP2A-B′ treatment altered the co-migration of pCyk4 (1–177) and Ect2 over gel filtration chromatography.

### Top-down MS

Purified recombinant Cyk4 (GS(1–177), ‘GS’ is resulted from TEV cleavage of the GST-tagged Cyk4) after phosphorylation by Plk1 was subjected to top-down MS analysis to identify the phosphorylation sites by direct infusion on Bruker 12T solariX XR Fourier transform ion cyclotron resonance MS (Bruker, Billerica, MA, USA). High-resolution MS spectrum reveals differential phosphorylation of Cyk4, including mono-, bis-, tris-, tetrakis- and pentakis-phosphorylated proteoforms. Tandem MS strategies with CID and ECD were utilized to characterize each phosphorylated proteoform for the phosphorylation localization. ECD was performed using 0.4–0.8 V d.c. bias and a 25–70 ms duration time. CID was performed on isolated charge states with 15–30 V d.c. bias. Up to 2000 transients were averaged per spectrum to ensure high-quality ECD/CID spectra. All Fourier transform ion cyclotron resonance spectra were processed with MASH Suite Pro [53] with THRASH algorithm, using a signal-to-noise threshold of 3.0 and minimum fit of 60% and validated manually. The resulting monoisotopic mass lists were further searched with MSalign+ algorithm embedded in MASH Suite Pro [39, 40, 53].

### Culture of mammalian cells

HeLa and 293T cells were cultured in Dulbecco’s modified Eagle’s medium (Gibco, Thermo Fisher Scientific, Waltham, MA, USA) with 10% fetal bovine serum (Hyclone, GE Healthcare, Boston, MA, USA), 100 U ml^−1^ penicillin and 100 μg ml^−1^ streptomycin in a humidified atmosphere at 37 °C with 5% CO_2_.

### Cloning of mammalian cell expression vector and transfection of plasmid and siRNA

The B′γ1-YFP fusion protein expression cassette was cloned into pCMX-pl2 mammalian cell recombinant expression vector. A unit of 2 μg of pCMX-pl2-B′γ1-YFP plasmid mixed with 100 pmol control or B′γ1 siRNA was transfected into 293T cells in 3.5 cm dishes with 50–80% confluence using Lipofectamine 2000 (Invitrogen). Cells were collected 48 h after transfection, 60 μg of whole cell extract was examined by western blot to detect B′γ1-YFP fusion protein using anti-GFP antibody (Cell Signaling, 2956, Cambridge, MA, USA) and actin loading control (C4, Millipore, Billerica, MA, USA). The blot was visualized using IRDye 680RD and IRdye 800CW and analyzed with Odyssey infrared imaging system (LI-Cor Biosciences, Lincoln, NE, USA). The control siRNA (Ambion by Thermo Fisher Scientific, 4390843) and B′-subunit-specific siRNAs were transfected into HeLa cells with 30–50% confluence using Lipofectamine RNAiMAX reagent (Invitrogen), 50 pmol for control, B′β, B′γ1 and B′ε siRNA per well in 12-well plates according to the manufacturer’s protocol. Cells were fixed 24 h after transfection for immunostaining. The siRNAs for PPP2R5B (B′β), PPP2R5C (B′γ1), and PPP2R5E (B′ε) are GGAGCACUGGAACCAAACCtt/GGUUUGGUUCCAGUGCUCCtt, GUUACGUCAGUGUUGCGUCtt/GACGCAACACUGACGUAACtt and GGAGCUUAUCACUCUUCCAtt/UGGAAGAGUGAUAAGCUCCtg, respectively (Ambion by Thermo Fisher Scientific).

### Co-immunoprecipitation

FLAG- or V5-tagged B′ substrates (FLAG-SENP6 (Addgene plasmid #18065), FLAG-USP53 (Addgene plasmid #22606), FLAG-GLI2 (Addgene plasmid #84920) and V5-CIP2A) and YFP-B′γ1 were co-transfected into 293T cells and cultured by similar procedures mentioned above. The transfection and overexpression efficiency of both proteins were monitored by western blot using antibodies that specifically recognize YFP-tag, FLAG-tag (M2, Sigma-Aldrich, St Louis, MO, USA) and V5-tag (Millipore, AB3792). The interaction between tagged-B′γ1 substrates and YFP-B′γ1 were recognized by co-immunoprecipitation using anti-FLAG and anti-V5 antibody immobilized on protein G magnetic beads (Invitrogen) before immunoprecipitate YFP-B′γ1 48 h after transfection. Cells were lysed in lysis buffer (50 mm Tris-HCl pH 8.0, 150 mm NaCl, 1 mm EDTA, 1 mm Dithiothreitol and 0.5% Triton X-100) and 400 μg of cell extracts were immunoprecipitated at 4 °C in lysis buffer for 8 h followed by western blot. A unit of 80 μg of whole-cell extracts were examined by western blot to examine the protein expression. The experiments were repeated three times and the representative results were shown.

### Immunofluorescent staining

Coverslips cultured with HeLa cells overnight in 12- or 24-well plates were washed three times in phosphate-buffered-saline (PBS), and fixed with three different methods: (1) 4% formaldehyde (Tousimis, Rockville, MD, USA) in PBS at RT for 10 min, followed by permeabilization with 0.2% Triton in PBS at RT for 10 min; (2) 100% Methanol at −20Â°C for 10 min; (3) 10% trichloracetic acid in PBS on ice for 10 min. After fixation, the coverslips were washed with PBS twice, and then blocked with blocking buffer, 0.2 m glycine, 2.5% fetal bovine serum, 0.1% Triton X-100 in PBS at RT for 1 h or at 4 °C overnight. For immunostaining of PP2Ac, PP2A A-subunit and B′γ1 regulatory subunit, PBS was replaced by MTSB buffer containing 100 mm K-pipes, pH 6.9, with KOH, 30%glycerol and 1 mm MgSO_4_.

The fixed cells were immune-stained for PP2Ac after formaldehyde fixation (rabbit polyclonal antibody, customer-made by Genemed Synthesis Inc., San Antonio, TX, USA, 1:200 dilution), for PP2A A-subunit after formaldehyde fixation (rabbit polyclonal antibody, Millipore 07–250, 1:200 dilution), for Cyk4 after methanol fixation (Millipore 5G5, 1:200 dilution), for Ect2 (G-4, 1:200 dilution, Santa Cruz, Dallas, TX, USA), for active RhoA after trichloracetic acid fixation (Santa Cruz 26C4, 1:200 dilution) and tubulin (rat monoclonal, Bio-Rad, YL1/2, 1:500 dilution). The fluorescently labeled secondary antibodies include AlexaFluor488 goat anti-rabbit, AlexaFluor594 goat anti-rabbit, AlexaFluor488 goat anti-mouse, AlexaFluor555 donkey anti-mouse and AlexaFluor594 donkey anti-rat (Thermo Fisher Scientific, 1:500–1000). DNA was stained briefly by DAPI 300 nm in PBS for 5 min, followed by wash with PBS 2–3 times. The stained coverslips were mounted onto slides using Vectashield mounting medium (Vector Laboratories, Burlingame, CA, USA), sealed with nail polish (Electron Microscopy Sciences, Hatfield, PA, USA) and examined by immunofluorescence microscopy using Zeiss Axio observer Inverted microscope (Zeiss, Oberkochen, Germany).

### Global search of intrinsic and phosphorylation-responsive B′-interaction motifs

Scripts written in R, a computer language and environment for statistical computing and graphics [54], were used to generate 9-mer motifs using MEME [55] from MEME suite [56] for identifying globally intrinsic and phosphorylation-responsive B′-interaction motifs using the sequences identified by ProP-PD for each B′ regulatory subunits, holoenzymes and the consolidated B′ family. The output consensus logos from MEME were then searched for all human protein sequences from www.uniprot.org [57, 58] using FIMO [59], with a *P*-value threshold set to include all of the associated training sequence motifs. The list was further filtered by *N*, a value calculated by numST+numDE-numRK. Each filtered motif was then annotated with secondary structures and domains from Uniprot [57, 58], average disorder calculations using IUPred [60, 61], previously identified phosphorylation sites from Uniprot [57, 58] and PhosphoSitePlus [3, 4], Netphos [62] phosphorylation predictions in the second, seventh, eighth and ninth position, and locations of the proteins containing the identified motifs at cellular super-complexes from the Microkits database [23]. Finally, gene ontology analysis of the identified proteins were performed by running GOSeq [63] using KEGG pathways [64].

### Cell cycle synchronization and detection of PP2A–Cyk4 interaction during anaphase in the presence and absence of Plk1 inhibitor

Thirty-six hours after HeLa cells were transfected with FLAG-Cyk4 and V5-CIP2A expression vectors, the transfected cells were arrested at anaphase with 100 μm monastrol for 14 h, followed by incubation with 200 nm BI-2536 or solvent control for 30 min. Cells were collected at the indicated time points after removal of monastrol to allow cell cycle progression. The interaction between FLAG-Cyk4 and PP2Ac were determined by co-immunoprecipitation using antibody that specifically recognizes FLAG-tag. PP2Ac associated with FLAG-Cyk4 and from cell lysate input were determined by western blot using customer-made antibody against PP2Ac.

## Figures and Tables

**Table 1:** Examples of predicted PP2A-B′γ1 substrates at large cellular complexes during mitosis and cytokinesis. Underlined sequences match the consensus motif.

*Uniprot*	*Gene names*	*Protein names*	*Sequence*	*Motif*	*D/E counts*^a^	*Location*
P51532	*SMARCA4*	Transcription activator BRG1	RAFLQAILEHEEQD	LxxIxExE	2	k
P52732	*KIF11*	Kinesin-like protein KIF11	LGSLTSIPENVSTH	LxxIxE	1	c; s
Q08379	*GOLGA2*	Golgin subfamily A member 2	PQPMPSIPEDLESR	MxxIxED	2	m
Q13177	*PAK2*	Serine/threonine-protein kinase PAK2	LKPLPSVPEEKKPR	LxxVxEE	2	c
Q14674	*ESPL1*	Extra spindle pole-like 1 protein	PEIMRTIPEEELTD	MxxIxEEE	3	c
Q16589	*CCNG2*	Cyclin-G2	VPELPTIPEGGCFD	LxxIxE	1	c
Q5VT06	*CEP350*	Centrosome-associated protein 350	RGSLESIAEHVDAS	LExIxE	2	c
Q92974	*ARHGEF2*	Rho guanine nucleotide exchange factor 2	FTRMQDIPEETESR	MxxIxEE	2	c; m; s
Q9Y2I6	*NLP*	Ninein-like protein	AERLQAIQEERARS	LxxIxEE	2	c
Q9Y2T1	*AXIN2*	Axin-2	CGYLPTLNEEEEWT	LxxLxEEE	3	c

Abbreviations: c, centrosome; k, kinetochore; m, midbody; PP2A, protein phosphatase 2A; s, spindle.

a Number of D or E at motif positions 2, 6, 7, 8 and 9.

**Table 2:** Examples of predicted motifs from proteins located at centrosome and midbody that upon phosphorylation might be recognized by PP2A-B′γ1. Underlined sequences match the consensus motif.

*Uniprot*	*Gene names*	*Protein names*	*Sequence*	*Predicted motif*	*Potential Pi sites*	*Location*	*Phospho-site*^a^
Q9C0D2	*CEP295*	Centrosomal protein of 295 kDa	SSSLSQVDESERFQ	LSpxVxESpE	2S7S	c	
Q9C0D2	*CEP295*	Centrosomal protein of 295 kDa	TKKLSQLGESELFA	LSpxLxESpE	2S7S	c	
Q9H1A4	*ANAPC1*	Anaphase-promoting complex subunit 1 (APC1)	VVLLSPVPELRDSS	LSpxVxE	2S	c; k	2S
O95835	*LATS1*	Serine/threonine-protein kinase LATS1	RQMLQEIRESLRNL	LxxIxESp	7S	c; m	7S
Q99996	*AKAP9*	A-kinase anchor protein 9	NLELQVLLESEKVR	LxxLxESpE	7S	c; m	
Q02224	*CENPE*	Centromere-associated protein E	RETLAKIQESQSKQ	LxxIxESp	7S	k; m; s	
Q08379	*GOLGA2*	Golgin subfamily A member 2	QEKLSELKETVELK	LSpxLxETp	2S7T	m	
Q8NF91	*SYNE1*	Nesprin-1	SSDLSTIQERMEEL	LSpxIxE	2S	m	
P42345	*MTOR*	Serine/threonine-protein kinase mTOR	SPGLTTLPEASDVG	LTpxlPE	2T	m; s	
Q9H0H5	*RACGAP1*	Rac GTPase-activating protein 1 (Cyk4)	NKRLSTIDESGSIL	LSpxIxESp	2S7S	m; s	2S7S

Abbreviations: c, centrosome; k, kinetochore; m, midbody; PP2A, protein phosphatase 2A; s, spindle.

a Phosphorylation sites listed in http://www.phosphosite.org.
