# Supplementary material for: PP2A-B′ holoenzyme substrate recognition, regulation and role in cytokinesis
Source: Cell Discov. 2017 Aug 8;3:17027–. doi: 10.1038/celldisc.2017.27 (PMC5586252; doi:10.1038/celldisc.2017.27)
Supplement: Supplementary Figures [file celldisc201727-s1.pdf]

PP2A-B' holoenzyme substrate recognition, regulation, and role in cytokinesis

Cheng-Guo Wu[1,2,8], Hui Chen[1,8], Feng Guo[1,8†], Vikash K Yadav[3,8], Sean J Mcilwain[4], Michael Rowse[1], Alka Choudhary[5], Ziqing Lin[6], Yitong Li[1], Tingjia Gu[1], Aiping Zheng[1‡], Qingge Xu[6], Woojong Lee[1,5], Eduard Resch[7], Benjamin Johnson[1], Ying Ge[6], Irene M Ong[4], Mark E. Burkard[5], Ylva Ivarsson[3,*], Yongna Xing[1,2,7,*]

[1]McArdle Laboratory for Cancer Research, Department of Oncology, University of Wisconsin at Madison, School of Medicine and Public Health, Madison, Wisconsin 53705, USA

[2]Biophysics program, University of Wisconsin at Madison, Wisconsin 53706, USA

[3]Department of Chemistry – BMC, Uppsala University, Uppsala 75123, Sweden

[4]Biostatistics and Medical Informatics, Wisconsin Institutes of Medical Research, University of Wisconsin at Madison, School of Medicine and Public Health, Madison, Wisconsin 53705, USA

[5]Department of Medicine, Hematology/Oncology, and UW Carbone Cancer Center University of Wisconsin at Madison, School of Medicine and Public Health, Madison, Wisconsin 53705, USA

[6]Department of Cell and Regenerative Biology and Human Proteomic Program, School of Medicine and Public Health, Madison, Wisconsin 53705, USA

[7]Fraunhofer Institute for Molecular Biology and Applied Ecology IME, Project Group Translational Medicine and Pharmacology TMP, Theodor-Stern-Kai 7, 60596 Frankfurt am Main, Germany

[8]These authors contributed equally to the project

[†]Currently Stanford University, California, USA

[‡]Currently University of Pittsburgh, Pennsylvania, USA

*To whom correspondence should be addressed:

Yongna Xing

E-mail: xing@oncology.wisc.edu;

Phone: 608-262-8376

Fax: 608-262-2824

Ylva Ivarsson

Email: Ylva.Ivarsson@kemi.uu.se

Phone: 018-471-4038

Supplemental materials

Figures S1-S6

Tables S1-S4 (in separate excel files)

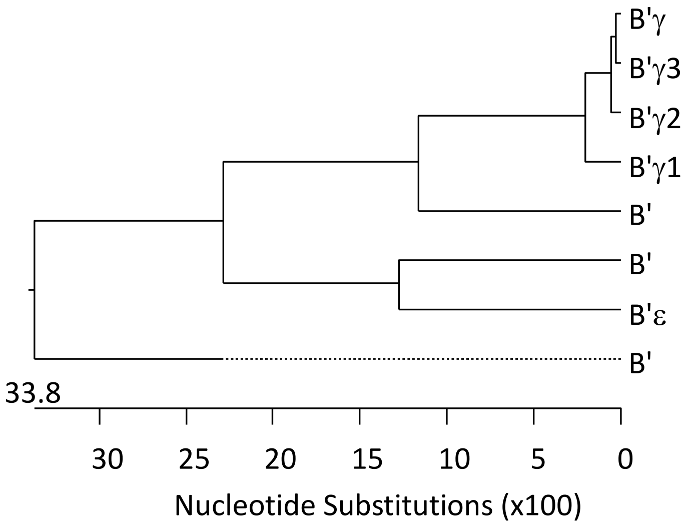

Figure S1. The phylogenetic tree of all the known members of B'-family PP2A regulatory subunits.

Peptide: "LEPIR***pS***EE" → B'γ1

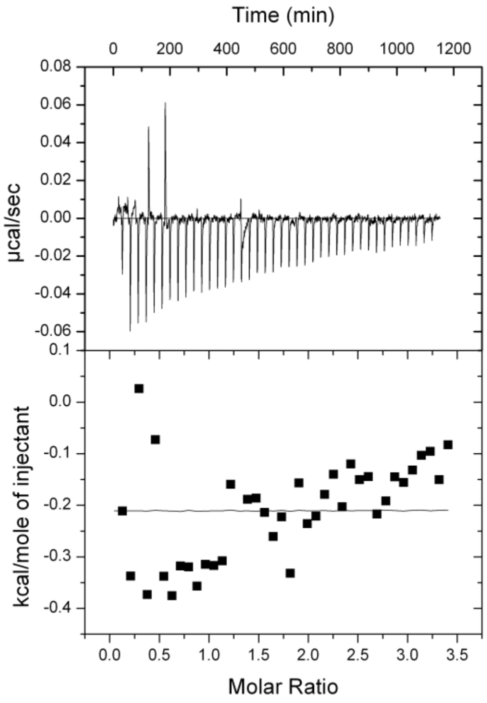

Figure S2. ITC measurement of the binding affinities between B'γ1 and synthetic peptides harboring S/T phosphorylation at positon 6 and 3 negatively charged residues at positions 2, 7, and 8 of B'-binding motifs. The change of the heat during peptide titrations are extremely low and the data analysis indicated no binding between the indicated peptide and B'γ1. Similar results were obtained for peptides LEPVRpSEE and LEPIRpTEE. pS and pT represent phosphorylated Serine and Threonine.

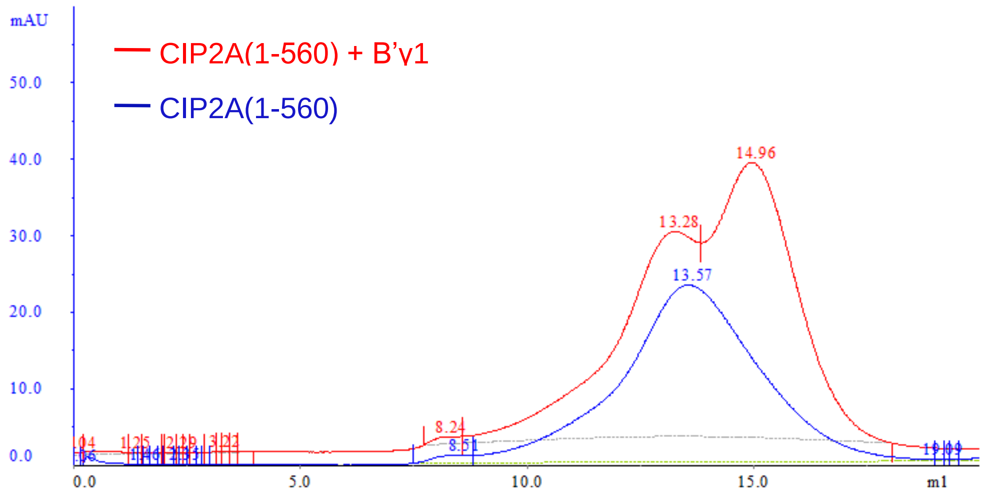

Figure S3. The N-terminal helix domain of CIP2A (encompassing residues 1-560) doesn't interact with B'γ1. Migration of CIP2A (1-560) alone and its mixture with slightly excess amount of B'γ1 over gel filtration chromatography. After correction of the void volume peak position (8.24+0.27=8.51ml), the peak of CIP2A (1-560) was barely shifted by the presence of B'γ1 (13.28+0.27=13.55~13.57ml). The lack of co-migration suggests that there is not interaction between CIP2A (1-560) and B'γ1.

Figure S4. Secondary structure prediction of examples shown in table1 by XtalPred[1]. Only regions that close to the predicted motifs of examples were shown.

Legends
Loop: loop secondary structure predicted by **PSIPRED**[2,3]
Helix: helix secondary structure predicted by **PSIPRED**
Strand: strand secondary structure predicted by **PSIPRED**
Disorder: disordered region predicted by **Disordered2**[2,3]
Dashed line box: regions of sequences containing putative motif for B' regulatory subunit binding

ATP-dependent helicase (SMARCA4)

RAFLQAILEHEEQD

```
....*.1210....*.1220....*.1230....*.1240....*.1250....*.1260....*.1270....*.1280....*.1290....*.1300
VLRLCTVNSVEEKILAAAKYKLNVDQKVIQAGMFDQKSSSHERRAFLQAILEHEEQDESRHCSTGSGSASFAHTAPPPAGVNPDLEEPPLKEEDEVPDDE

....*.1310....*.1320....*.1330....*.1340....*.1350....*.1360....*.1370....*.1380....*.1390....*.1400
TVNQMIARHEEEFDLFMRMDLDRRREEARNPKRKPRLMEEDELPSWIIKDDAEVERLTCEEEEEKMFGRGSRHRKEVDYSDSLTEKQWLKKITGKDIHDT
```

Kinesin-like protein (KIF11) ; Kinesin-related motor protein (Eg5)

LGSLTSIPENVSTH

```
....*..510....*..520....*..530....*..540....*..550....*..560....*..570....*..580....*..590....*..600
NTVEETTKDVSGLHSKLDRKKAVDQHNAEAQDIFGKNLNSLFNNMEELIKDGSSKQKAMLEVHKTLFGNLLSSSVSALDTITTVALGSLTSIPENVSTHV

....*..610....*..620....*..630....*..640....*..650....*..660....*..670....*..680....*..690....*..700
SQIFNMILKEQSLAAESKTVLQELINVLKTDLLSSLEMILSPTVVSILKINSQLKHIFKTSLTVADKIEDQKKELDGFLSILCNNLHELQENTICSLVES
```

Golgin subfamily A member 2 (GOLGA2)

PQPMPSIPEDLESR

```
....*..610....*..620....*..630....*..640....*..650....*..660....*..670....*..680....*..690....*..700
KSQEAQSLQQQRDQYLGHLQQYVAAYQQLTSEKEVLHNQLLLQTQLVDQLQQQEAQGKAVAEMARQELQETQERLEAATQQNQQLRAQLSLMAHPGEGDG

....*..710....*..720....*..730....*..740....*..750....*..760....*..770....*..780....*..790....*..800
LDREEEEDEEEEEEEAVAVPQPMPSIPEDLESREAMVAFFNSAVASAEEEQARLRGQLKEQRVRCRRLAHLLASAQKEPEAAAPAPGTGGDSVCGETHRA

....*..810....*..820....*..830....*..840....*..850....*..860....*..870....*..880....*..890....*..900
LQGAMEKLQSRFMELMQEKADLKERVEELEHRCIQLSGETDTIGEYIALYQSQRAVLKERHREKEEYISRLAQDKEEMKVKLLELQELVLRLVGDRNEWH
```

p21-activated kinase 2 (PAK2)

LKP<u>LPSVPEE</u>KKPR

```
1...*...10....*...20....*...30....*...40....*...50....*...60....*...70....*...80....*...90....*..100
MSDNGELEDKPPAPPVRMSSTIFSTGGKDPLSANHSLKPLPSVPEEKKPRHKIISIFSGTEKGSKKKEKERPEISPPSDFEHTIHVGFDAVTGEFTGMPE

....*..110....*..120....*..130....*..140....*..150....*..160....*..170....*..180....*..190....*..200
QWARLLQTSNITKLEQKKNPQAVLDVLKFYDSNTVKQKYLSFTPPEKDGFPSGTPALNAKGTEAPAVVTEEEDDDEETAPPVIAPRPDHTKSIYTRSVID
```

Extra Spindle poles-like 1 protein (Separase)

PEI<u>MRTIPEEE</u>LTD

```
....*.1410....*.1420....*.1430....*.1440....*.1450....*.1460....*.1470....*.1480....*.1490....*.1500
LEDPVSAEAWLAEEPKRRGTASRGRGRARKGLSLKTDAVVAPGSAPGNPGLNGRSRRAKKVASRHCEERRPQRASDQARPGPEIMRTIPEEELTDNWRKM

....*.1510....*.1520....*.1530....*.1540....*.1550....*.1560....*.1570....*.1580....*.1590....*.1600
SFEILRGSDGEDSASGGKTPAPGPEAASGEWELLRLDSSKKKLPSPCPDKESDKDLGPRLRLPSAPVATGLSTLDSICDSLSVAFRGISHCPPSGLYAHL
```

Cyclin-G2 (CCNG2)

VPE<u>LPTIPE</u>GGCFD

```
....*..110....*..120....*..130....*..140....*..150....*..160....*..170....*..180....*..190....*..200
IGVCSFLLAARIVEEDCNIPSTHDVIRISQCKCTASDIKRMEKIISEKLHYELEATTALNFLHLYHTIILCHTSERKEILSLDKLEAQLKACNCRLIFSK

....*..210....*..220....*..230....*..240....*..250....*..260....*..270....*..280....*..290....*..300
AKPSVLALCLLNLEVETLKSVELLEILLLVKKHSKINDTEFFYWRELVSKCLAEYSSPECCKPDLKKLVWIVSRRTAQNLHNSYYSVPELPTIPEGGCFD

....*..310....*..320....*..330....*..340....
ESESEDSCEDMSCGEESLSSSPPSDQECTFFFNFKVAQTLCFPS
```

Centrosome-associated protein 350 (CEP350)

RGS<u>LESIAE</u>HVDAS

```
....*.2110....*.2120....*.2130....*.2140....*.2150....*.2160....*.2170....*.2180....*.2190....*.2200
IKKTEAELSQDLETSPTAKPQIKTLSSASEKPKIKPLTPLHRSETAKNWKSLTESERSRGSLESIAEHVDASLSGSERSVSERSLSAYAKRVNEWDSRTE

....*.2210....*.2220....*.2230....*.2240....*.2250....*.2260....*.2270....*.2280....*.2290....*.2300
DFQTPSPVLRSSRKIREESGDSLENVPALHLLKELNATSRILDMSDGKVGESSKKSEIKEIEYTKLKKSKIEDAFSKEGKSDVLLKLVLEQGDSSEILSK
```

Rho guanine nucleotide exchange factor 2 (ARHGEF2)

FTR<u>MQDIPEE</u>TESR (967-974)

```
....*..810....*..820....*..830....*..840....*..850....*..860....*..870....*..880....*..890....*..900
TELALLQRQHALLQEELRRCRRLGEERATEAGSLEARLRESEQARALLEREAEEARRQLAALGQTEPLPAEAPWARRPVDPRRRSLPAGDALYLSFNPPQ

....*..910....*..920....*..930....*..940....*..950....*..960....*..970....*..980....*
PSRGTDRLDLPVTTRSVHRNFEDRERQELGSPEERLQDSSDPDTGSEEEGSSRLSPPHSPRDFTRMQDIPEETESRDGEAVASES
```

Ninein-like protein (NLP)

AERLQAIQEERARS (975-982)

```
....*..910....*..920....*..930....*..940....*..950....*..960....*..970....*..980....*..990....*.1000
GPSERWSRMQPCGVDGDIVPKEPEPFGASAAGLEQPGARELPLLGTERDASQTQPRMWEPPLRPAASCRGQAERLQAIQEERARSWSRGTQEQASEQQAR

....*.1010....*.1020....*.1030....*.1040....*.1050....*.1060....*.1070....*.1080....*.1090....*.1100
AEGALEPGCHKHSVEVARRGSLPSHLQLADPQGSWQEQLAAPEEGETKIALEREKDDMETKLLHLEDVVRALEKHVDLRENDRLEFHRLSEENTLLKNDL
```

Axin-2 (AXIN2)

CGYLPTLNEEEEWT (225-232)

```
....*..110....*..120....*..130....*..140....*..150....*..160....*..170....*..180....*..190....*..200
EKCVDTLDFWFACNGFRQMNLKDTKTLRVAKAIYKRYIENNSIVSKQLKPATKTYIRDGIKKQQIDSIMFDQAQTEIQSVMEENAYQMFLTSDIYLEYVR

....*..210....*..220....*..230....*..240....*..250....*..260....*..270....*..280....*..290....*..300
SGGENTAYMSNGGLGSLKVVCGYLPTLNEEEEWTCADFKCKLSPTVVGLSSKTLRATASVRSTETVDSGYRSFKRSDPVNPYHIGSGYVFAPATSANDSE
```

Figure S5. Secondary structure prediction of examples shown in table2 by XtalPred[1]. Only regions that close to the predicted motifs of examples were shown.

Centrosomal protein of 295 kDa (CEP295)

SSSLSQVDESERFQ

TKKLSQLGESELFA

```
....*.1110....*.1120....*.1130....*.1140....*.1150....*.1160....*.1170....*.1180....*.1190....*.1200
SSPVVVQHSVASQASAKAEPRRIQELYLSEKENVGPSCHLIIPTFQDKSLSFPQHSLAQQENLTILQEQSQIQRVILGAKEGTQEFVHTESELEKRISSE

....*.1210....*.1220....*.1230....*.1240....*.1250....*.1260....*.1270....*.1280....*.1290....*.1300
QTGTSSSLSQVDESERFQECISIKSDSTIPLSHPKIPRCQERLLRVSQHMLPLQDNLEEHQAWLDTEKEAFHFSQKTQENTSSEQTGSSSFIPQLVQLSF

....*.2310....*.2320....*.2330....*.2340....*.2350....*.2360....*.2370....*.2380....*.2390....*.2400
DNKNETCRVLDINPQVEETDSRLCVRTVEMGTSIQAPYSLTTQNEKYFENSAETDIPKITKKLSQLGESELFASSGSFSLQSSIPVWETETGHGIMEEPE

....*.2410....*.2420....*.2430....*.2440....*.2450....*.2460....*.2470....*.2480....*.2490....*.2500
LTLISTTDTSIAEMDFANLTLEEKSENEAKCFFQVSEFLPLVSATEASDYPAVSELSIEKPRTASTETPRRLTPVPGSLQEAFIKRKKSFMERSHQRQKE
```

Anaphase-promoting complex subunit 1 (APC-1)

VVLLSPVPELRDSS (locates within a long loop and between two disordered regions; potentially a disordered structure)

```
....*..510....*..520....*..530....*..540....*..550....*..560....*..570....*..580....*..590....*..600
TGVVRVGKVFIPGLPAPSLTMSNTMPRPSTPLDGVSTPKPLSKLLGSLDEVVLLSPVPELRDSSKLHDSLYNEDCTFQQLGTYIHSIRDPVHNRVTLELS

....*..610....*..620....*..630....*..640....*..650....*..660....*..670....*..680....*..690....*..700
NGSMVRITIPEIATSELVQTCLQAIKFILPKEIAVQMLVKWYNVHSAPGGPSYHSEWNLFVTCLMNMMGYNTDRLAWTRNFDFEGSLSPVIAPKKARPSE
```

Centromere protein F (CENP-F)

QELLQRVETSEGLN

ENELSRIRSEKASI

```
....*.1710....*.1720....*.1730....*.1740....*.1750....*.1760....*.1770....*.1780....*.1790....*.1800
DLNLDIEKITETGAVKPTGECSGEQSPDTNYEPPGEDKTQGSSECISELSFSGPNALVPMDFLGNQEDIHNLQLRVKETSNENLRLLHVIEDRDRKVESL

....*.1810....*.1820....*.1830....*.1840....*.1850....*.1860....*.1870....*.1880....*.1890....*.1900
LNEMKELDSKLHLQEVQLMTKIEACIELEKIVGELKKENSDLSEKLEYFSCDHQELLQRVETSEGLNSDLEMHADKSSREDIGDNVAKVNDSWKERFLDV

....*.1910....*.1920....*.1930....*.1940....*.1950....*.1960....*.1970....*.1980....*.1990....*.2000
ENELSRIRSEKASIEHEALYLEADLEVVQTEKLCLEKDNENKQKVIVCLEEELSVVTSERNQLRGELDTMSKKTTALDQLSEKMKEKTQELESHQSECLH

....*.2010....*.2020....*.2030....*.2040....*.2050....*.2060....*.2070....*.2080....*.2090....*.2100
CIQVAEAEVKEKTELLQTLSSDVSELLKDKTHLQEKLQSLEKDSQALSLTKCELENQIAQLNKEKELLVKESESLQARLSESDYEKLNVSKALEAALVEK
```

Serine/threonine-protein kinase LATS1 （LATS1）

RQMLQEIRESLRNL

```
1...*...10....*...20....*...30....*...40....*...50....*...60....*...70....*...80....*...90....*..100
MKRSEKPEGYRQMRPKTFPASNYTVSSRQMLQEIRESLRNLSKPSDAAKAEHNMSKMSTEDPRQVRNPPKFGTHHKALQEIRNSLLPFANETNSSRSTSE
```

A-kinase anchor protein 9 (AKAP-9)

NLELQVLLESEKVR

```
....*.3210....*.3220....*.3230....*.3240....*.3250....*.3260....*.3270....*.3280....*.3290....*.3300
KDKTDEVHLLNDTLASEQKKSRELQWALEKEKAKLGRSEERDKEELEDLKFSLESQKQRNLQLNLLLEQQKQLLNESQQKIESQRMLYDAQLSEEQGRNL

....*.3310....*.3320....*.3330....*.3340....*.3350....*.3360....*.3370....*.3380....*.3390....*.3400
ELQVLLESEKVRIREMSSTLDRERELHAQLQSSDGTGQSRPPLPSEDLLKELQKQLEEKHSRIVELLNETEKYKLDSLQTRQQMEKDRQVHRKTLQTEQE

....*.3410....*.3420....*.3430....*.3440....*.3450....*.3460....*.3470....*.3480....*.3490....*.3500
ANTEGQKKMHELQSKVEDLQRQLEEKRQQVYKLDLEGQRLQGIMQEFQKQELEREEKRESRRILYQNLNEPTTWSLTSDRTRNWVLQQKIEGETKESNYA
```

Centromere-associated protein E (CENP-E)

RETLAKIQESQSKQ

```
....*.1310....*.1320....*.1330....*.1340....*.1350....*.1360....*.1370....*.1380....*.1390....*.1400
TQETMNELELLTEQSTTKDSTTLARIEMERLRLNEKFQESQEEIKSLTKERDNLKTIKEALEVKHDQLKEHIRETLAKIQESQSKQEQSLNMKEKDNETT

....*.1410....*.1420....*.1430....*.1440....*.1450....*.1460....*.1470....*.1480....*.1490....*.1500
KIVSEMEQFKPKDSALLRIEIEMLGLSKRLQESHDEMKSVAKEKDDLQRLQEVLQSESDQLKENIKEIVAKHLETEEELKVAHCCLKEQEETINELRVNL
```

Golgin subfamily A member 2 (GOLGA2)

QEKLSELKETVELK

```
....*..510....*..520....*..530....*..540....*..550....*..560....*..570....*..580....*..590....*..600
EERLLELERAAELWGEQAEARRQILETMQNDRTTISRALSQNRELKEQLAELQSGFVKLTNENMEITSALQSEQHVKRELGKKLGELQEKLSELKETVEL

....*..610....*..620....*..630....*..640....*..650....*..660....*..670....*..680....*..690....*..700
KSQEAQSLQQQRDQYLGHLQQYVAAYQQLTSEKEVLHNQLLLQTQLVDQLQQQEAQGKAVAEMARQELQETQERLEAATQQNQQLRAQLSLMAHPGEGDG
```

Nuclear envelope spectrin repeat protein 1 (Syne-1)

KLFLSELQTTSEIS

```
....*.1710....*.1720....*.1730....*.1740....*.1750....*.1760....*.1770....*.1780....*.1790....*.1800
ALQNEVVSQASFYSKLLQLKESLFSVASKDDVKMMKLHLEQLDERWRDLPQIINKRINFLQSVVAEHQQFDELLLSFSVWIKLFLSELQTTSEISIMDHQ

....*.1810....*.1820....*.1830....*.1840....*.1850....*.1860....*.1870....*.1880....*.1890....*.1900
VALTRHKDHAAEVESKKGELQSLQGHLAKLGSLGRAEDLHLLQGKAEDCFQLFEEASQVVERRQLALSHLAEFLQSHASLSGILRQLRQTVEATNSMNKN
```

Serine/threonine-protein kinase mTOR (MTOR)

SPGLTTLPEASDVG

```
....*..410....*..420....*..430....*..440....*..450....*..460....*..470....*..480....*..490....*..500
AFRPSAFTDTQYLQDTMNHVLSCVKKEKERTAAFQALGLLSVAVRSEFKVYLPRVLDIIRAALPPKDFAHKRQKAMQVDATVFTCISMLARAMGPGIQQD

....*..510....*..520....*..530....*..540....*..550....*..560....*..570....*..580....*..590....*..600
IKELLEPMLAVGLSPALTAVLYDLSRQIPQLKKDIQDGLLKMLSLVLMHKPLRHPGMPKGLAHQLASPGLTTLPEASDVGSITLALRTLGSFEFEGHSLT
```

Rac GTPase-activating protein 1 (CYK4)

NKRLSTIDESGSIL

```
....*..110....*..120....*..130....*..140....*..150....*..160....*..170....*..180....*..190....*..200
QLIREMLMCDTSGSIQLSEEQKSALAFLNRGQPSSSNAGNKRLSTIDESGSILSDISFDKTDESLDWDSSLVKTFKLKKREKRRSTSRQFVDGPPGPVKK

....*..210....*..220....*..230....*..240....*..250....*..260....*..270....*..280....*..290....*..300
TRSIGSAVDQGNESIVAKTTVTVPNDGGPIEAVSTIETVPYWTRSRRKTGTLQPWNSDSTLNSRQLEPRTETDSVGTPQSNGGMRLHDFVSKTVIKPESC
```

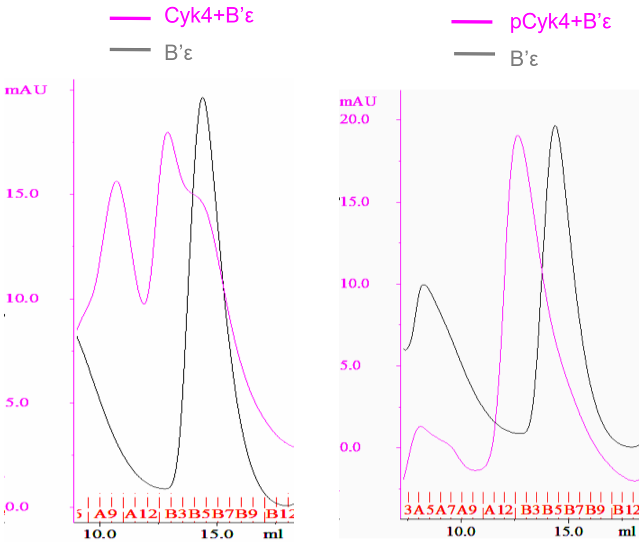

Figure S6. Gel filtration chromatography of B'ε alone and its mixture with Cyk4 (1-177) (left panel) and pCyk4 (1-177) (right panel). While B'ε co-migrates with pCyk4 (1-177), but not Cyk4 (1-177).

REFERENCE:

1. Slabinski L, Jaroszewski L, Rychlewski L, et al., XtalPred: a web server for prediction of protein crystallizability. *Bioinformatics*, 2007; **23(24)**:3403-5.

2. Jones DT. Protein secondary structure prediction based on position-specific scoring matrices. *J. Mol. Biol.* 1999; **292**:195-202.

3. Buchan DWA, Minneci F, Nugent TCO, et al., Scalable web services for the PSIPRED Protein Analysis Workbench. *Nucleic Acids Research.* 2013; **41 (W1)**: W340-W348.
